# Impact of responsive insertion technology (RIT) on reducing discomfort during colonoscopy: randomized clinical trial

**DOI:** 10.1007/s00464-016-5226-x

**Published:** 2016-09-08

**Authors:** Artur Pasternak, Miroslaw Szura, Rafal Solecki, Maciej Matyja, Antoni Szczepanik, Andrzej Matyja

**Affiliations:** 1 First Chair of General, Oncological and Gastrointestinal Surgery, Jagiellonian University Medical College, 40th Kopernika St., 31-501 Krakow, Poland; 2 Department of Anatomy, Jagiellonian University Medical College, 12th Kopernika St., 31-034 Krakow, Poland; 3 Department of Experimental and Clinical Surgery, Jagiellonian University Medical College, 12th Michalowskiego St., 31-126 Krakow, Poland; 4 Second Chair of General Surgery, Jagiellonian University Medical College, 21st Kopernika St., 31-501 Krakow, Poland

**Keywords:** Colonoscopy, Colorectal cancer, Responsive insertion technology

## Abstract

**Background:**

In many countries, colonoscopies for colorectal cancer screening are performed without sedation due to the cost. Changes in the structure of the endoscopes are designed to facilitate the colonoscopic examination, reduce the duration of the procedure, and improve the imaging of the intestinal lumen. The variable stiffness of the endoscope and the recently introduced responsive insertion technology (RIT) are features aimed at easing colonoscope insertion and reducing the discomfort and pain during the examination. The aim of the study is to analyze whether the new RIT system can improve the practice of colonoscopy under no anesthesia with respect to the widely available variable stiffness colonoscopes.

**Materials and methods:**

This analysis included 647 patients who underwent complete colonoscopy in the screening program. All colonoscopies were performed without sedation. Olympus series 180 and 190 endoscopes equipped with a magnetic positioning system were used. Group I included patients who were examined using endoscopes equipped with responsive insertion technology (RIT), and group II included patients who were examined using conventional variable stiffness colonoscopies. The main objective was to evaluate the cecal intubation time, the number of loops, the requirement to apply manual pressure to different areas of the abdomen and the degree of discomfort and pain expressed on a visual analogue scale (VAS). ClinicalTrials.gov number, NCT01688557.

**Results:**

Group I consisted of 329 patients, and group II included 318 patients. The mean age of the patients was 58.4 years (SD ± 4.21). Both groups were compared in terms of age, sex, and BMI. The mean cecal intubation time was 209 s in group I and 224 s in group II (*p* < 0.05). Increased loop formation was observed upon endoscope insertion in group II (1.7 vs. 1.35) (*p* < 0.05) and required more manual pressure to the abdomen (2.2 vs. 1.7) (*p* = 0.001). In group I, less discomfort and pain, as graded on a VAS (2.3 vs. 2.6), were noted.

**Conclusions:**

The implementation of RIT reduced of the cecal intubation time. The modified structure of the endoscope rendered the colonoscopic examination easier by reducing loop formation upon insertion with a subsequently reduced rate of auxiliary maneuvers.

Colorectal cancer (CRC) is the third most common cancer and the fourth leading cause of cancer-related death worldwide [1]. Since 2009, mounting evidence from observational studies has demonstrated that colonoscopy screening is associated with reductions in both CRC incidence and mortality [2–5]. Most cases of CRC arise from adenoma via a process known as the adenoma–carcinoma sequence and are therefore amenable to screening and early treatment [6, 7]. Approximately 98 % of all colonoscopies in the USA are performed with sedation [8]. Traditionally, sedation involves a benzodiazepine and an opioid. Recently, propofol has been utilized as an alternative option for sedation due to its rapid induction of sedation, faster recovery, lack of active metabolites, and equivalent levels of amnesia. However, in many other countries (e.g., Poland), colonoscopies for CRC screening are performed without sedation due to the costs. The structure of endoscopes has been altered to facilitate feasibility of the examination, reduce the time of its duration, and diminish patient discomfort during examination. Responsive insertion technology (RIT) is a unique combination of three technologies: passive bending (PB), high-force transmission (HFT), and variable stiffness. These technologies work together to improve ease of insertion and operator control, which may help to minimize patient discomfort and enhance procedural efficiency.

PB helps colonoscopes move through acute bends in the colon because the passive bending section is located between the insertion tube and the conventional bending section of the endoscope. When the scope meets resistance, the pressure is redistributed such that the insertion tube automatically bends to adjust to the contours of the colon, thereby potentially decreasing patient discomfort and providing rapid insertion to the cecum.

HFT provides improved operator control for pushing and twisting maneuvers. Whenever the scope is pushed forward or rotated, the pushing force or rotational torque is transmitted in a 1:1 manner down the length of the insertion tube. Thus, the scope reacts more sensitively to physician handling and is easier to maneuver within the colon. This technology features an insertion tube that better transmits the pushing force and torque by reducing the loss of force at the loop, thus helping the device pass the sigmoid colon with less pushing force and torque.

Variable stiffness allows the flexibility of scopes to be incrementally altered by manipulating a flexibility adjustment ring that ranges from 0 to 3. This innovative feature allows the variable stiffness colonoscope (VSC) to be adjusted on a case-by-case basis to meet the unique anatomical needs of the patient and the physician’s handling preferences.

The aim of the study was to analyze whether the new RIT system can improve the practice of colonoscopy under no anesthesia with respect to the widely available variable stiffness colonoscopes.

## Materials and methods

**Fig. 1:**
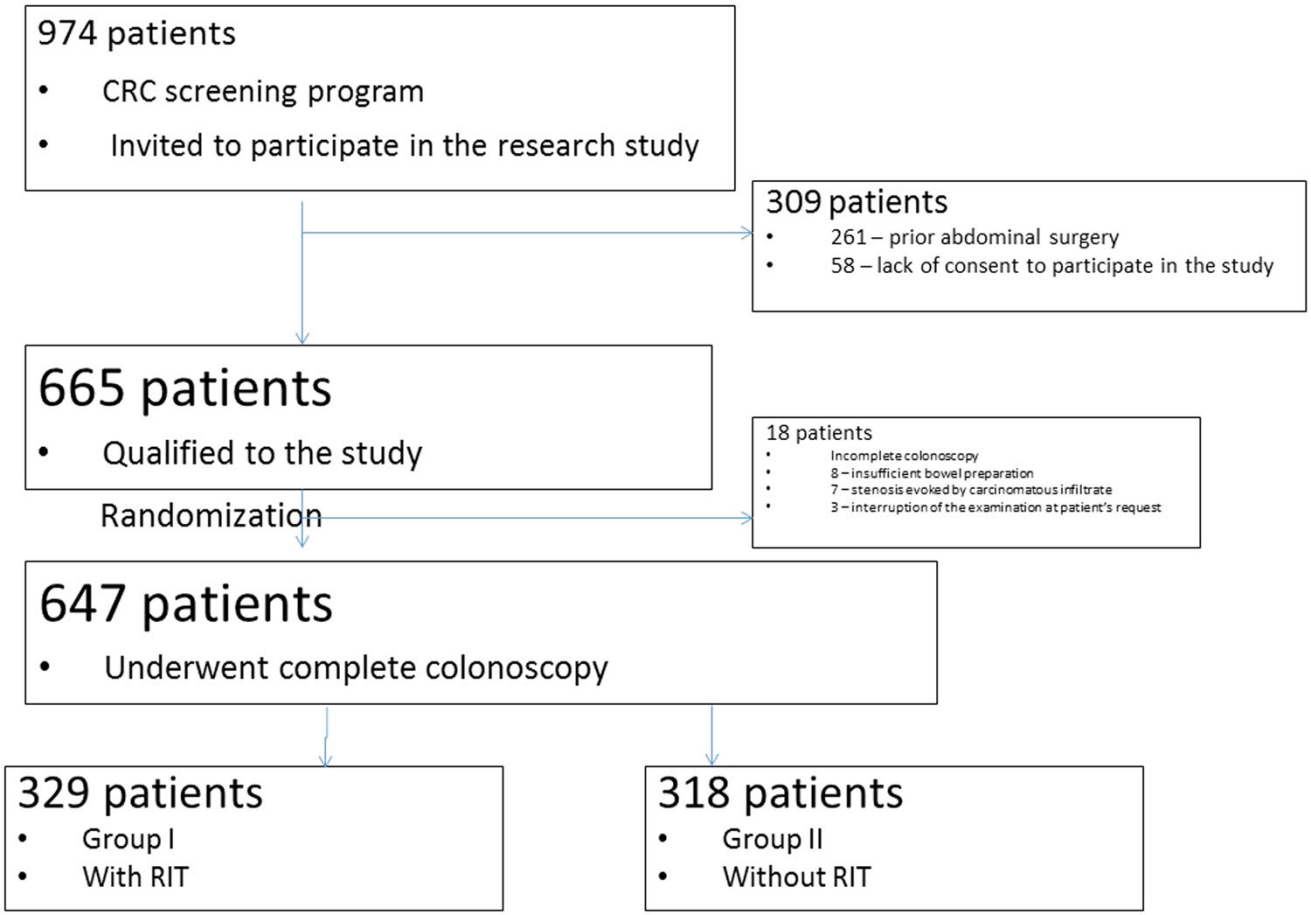
Consort diagram of patient enrollment

The analysis was performed between 2014 and 2015 at the Endoscopy Unit in Krakow as a part of a national colorectal cancer-screening program, which was financed by the Polish Ministry of Health. The study was approved by the local ethics committee and was conducted in accordance with the principles of the Declaration of Helsinki. Polish citizens aged 50–65 or 40–65 with a history of abdominal cancer in a first-degree relative took part in the analysis. Inclusion criteria were that patients were between 40 and 65 years of age, able to provide informed consent, whose indication for colonoscopy was colorectal cancer screening, and for whom this was a first or follow-up colonoscopy (Fig. 1). We excluded all patients with suspected significant gastrointestinal bleeding, previous abdominopelvic surgical history, previous colonic resections, known inflammatory bowel disease, or specific conditions that made it theoretically more desirable to use a specific colonoscope (e.g., stenosis, major bleeding), patients with a high anesthetic risk (ASA-4), pregnant women, and patients who were unable to provide informed consent.

Six hundred and sixty-five consecutive endoscopy unit outpatients who were scheduled to undergo colonoscopy screening for CRC were invited to participate in this study upon arrival for their appointment. Eighteen patients with an incomplete colonoscopy due solely to inadequate preparation or sedation were excluded (Fig. 1).

All patients were given the same bowel preparation guidelines based on the oral ingestion of liquid propulsive agents (i.e., 420 g of polyethylene glycol (PEG) in 4 L of water taken in 4 doses every 6 h one day before the colonoscopy). Bowel cleansing quality was graded at the end of the procedure according to the Boston bowel preparation scale.

All colonoscopies were performed by 7 experienced endoscopists (≥1000 colonoscopies), who had previously dealt with endoscopes equipped with RIT and possessed comparable experience in the use of this technology. All endoscopists were assisted by nurses who were responsible for applying manual pressure to different areas of the abdomen to facilitate endoscope insertion. All colonoscopies were performed without sedation. There was no technical possibility to blind the type of endoscope because of their completely different appearance, and clothing of endoscope in a sleeve camouflage would have hampered the performance of colonoscopy thus affecting the study results.

Olympus series 180 and 190 endoscopes equipped with magnetic positioning system were used. Group I included 329 patients who were examined using variable stiffness endoscopes equipped with RIT (Olympus CF-HQ190L, Olympus Optical Co. Ltd, Tokyo, Japan), and group II included 318 patients who were examined using conventional variable stiffness endoscopes (Olympus CF-H180DL, Olympus Optical Co. Ltd, Tokyo, Japan). The mean age of the patients was 58.4 years (SD ± 4.21). Patients were randomly assigned to two groups as described below. Randomization took place at the endoscopy unit at the study center. A computer-generated list was used for randomization. The randomization sequence was created by the R package “blockrand” with a 1:1 allocation using randomly varying block sizes. To allocate a patient to either the RIT or standard group, a sealed envelope was opened and the randomization card taken out before endoscopy. The endoscopy team did not take part in the randomization allocation process.

The main objectives were to evaluate the cecal intubation (CI) time, the rate of loop formation, the requirement of applying manual pressure to different areas of the abdomen, and degree of discomfort and pain expressed on a visual analogue scale (VAS). Cecal intubation was defined as the time of the insertion of the colonoscope tip to a point proximal to the appendiceal orifice. Loops were identified on the magnetic positioning system display during colonoscopic examination. Additionally, following the colonoscopic examination, the pain perceived by the patient was recorded using a VAS for pain of 0–10. On that scale, the absence of pain corresponds to 0, and the maximum bearable pain corresponds to 10. This parameter was collected by the nursing staff immediately after the colonoscopy (evaluation of intraprocedural pain) and again 15 and 60 min after the colonoscopy (evaluation of postprocedural pain).

## Statistics

Continuous variables are expressed as the mean ± SD. Categorical variables were expressed as frequencies and percentages. Differences between the groups of patients (RIT group vs conventional group) were detected using an independent *t* test or Mann–Whitney *U* test for continuous data and the Chi-square test or the Fisher’s exact test for categorical data, as appropriate. Univariate and multivariate linear regression models were used to identify factors affecting VAS pain scores during endoscope insertion. Multivariate linear regression with stepwise selection was applied; variables that did not improve the model fit at *p* < 0.05 were discarded. A *p*-value <0.05 was considered to indicate a statistically significant difference between groups. All statistical evaluations were performed using Statistica version 12 (StatSoft, Tulsa, OK, USA).

## Results

Both groups of patients were compared in terms of age, sex, and BMI. No differences in the distribution of sex, age, and BMI were observed between the groups of patients assigned to the novel RIT or conventional endoscope groups (Table 1).

**Table 1:** Patients characteristics

Group	Sex	*n*	Mean age	Age SD±	BMI min	BMI max	Mean BMI	BMI SD±
I	F	220	58.86	4.21	17	44	26.44	4.58
	M	109	58.18	4.15	21	42	28	3.81
II	F	224	58.25	4.20	18	40	26.26	4.16
	M	94	57.94	4.30	15	42	27.43	4.29
	*p* = 0.329		*p* = 0.146		*p* = 0.306			

No complications were observed in any of the procedures included in the study. All patients recovered and were discharged from the endoscopy unit. The complete cecal intubation rate was 100 % in both groups. The cecal intubation time was significantly reduced in the RIT endoscope group (group I: mean 209 s, SD 93.75 s) compared with the conventional endoscope group (group II: mean 224 s, SD 103.07 s) (*p* < 0.05) (Table 2).

**Table 2:** Cecal intubation time

Group	Sex	Min. cecal intubation time (s)		Max. cecal intubation time (s)	
I	M	70	50	480	520
	F	50		520	
II	M	60	50	610	620
	F	50		620	

Group	Sex	Mean cecal intubation time (s)		SD±	
I	M	221.72	209.29	111.09	93.75
	F	224.62		99.76	
II	M	198.79	223.76	100.37	103.07
	F	214.49		90.07	

We evaluated the number of loops encountered during colonoscopy. The number of undesired loops in the shaft of a flexible scope was significantly reduced when the RIT endoscope was used (group I: 1.30, SD 1.00 vs. group II: 1.70, SD 1.10) (*p* < 0.05) (Tables 3).

**Table 3:** Comparison of loop formations, number of manual compressions to the abdomen, and changes in patient position during endoscope insertion between two analyzed groups

Group	Sex	Loop formations								Number of manual compressions							
		Min		Max		Mean		SD±		Min		Max		Mean		SD±	
I	M	0	0	4	4	1.4	1.3	1.1	1.0	0	0	6	6	1.77	1.67	1.07	1.05
	F	0		4		1.2		1.0		0		5		1.49		0.98	
II	M	0	0	5	5	1.7	1.7	1.1	1.1	0	0	4	5	2.19	2.17	1.14	1.11
	F	0		5		1.6		1.1		0		5		2.14		1.05	
		*p* < 0.05								*p* < 0.05							

Group	Sex	Changes in patient position							
		Min		Max		Mean		SD±	
I	M	0	0	2	2	0.19	0.27	0.46	0.53
	F	0		2		0.31		0.55	
II	M	0	0	4	4	0.44	0.46	0.78	0.73
	F	0		4		0.46		0.71	
		*p* < 0.05							

Significant differences were also noted in the need for the application of manual pressure to the abdomen and the need to change the patient’s position. The total frequency of abdominal compressions applied by nurses during endoscopic insertion was reduced in group I (1.67, SD 1.05 vs. 2.17, SD 1.11) (Table 3).

Similar findings were noted concerning the need to change a patient’s position (0.27, SD 0.53 vs. 0.46, SD 0.73) (Table 3).

Abdominal pain was assessed using a 10-point VAS. We observed a significant trend of reduced pain in patients in whom colonoscopy was performed with the RIT system (Table 4).

**Table 4:** VAS pain score (at 1, 15 and 60 min after colonoscopy)

Group	Sex	Mean VAS (1 min)		VAS (1 min) SD±		Mean VAS (15 min)		VAS (15 min) SD±		Mean VAS (1 h)		VAS (1 h) SD±	
I	M	1.92	2.33	0.88	1.12	1.88	2.06	1.19	1.21	1.38	1.39	0.73	0.66
	F	2.53		1.17		2.15		1.21		1.39		0.62	
II	M	2.26	2.55	1.15	1.12	1.88	2.14	1.19	1.20	1.34	1.37	0.58	0.57
	F	2.67		1.22		2.25		1.20		1.37		0.56	

In group I, patients reported less intraprocedural pain during colonoscopic examination (2.33, SD 1.12 vs 2.55, SD 1.12) and less postprocedural pain registered 15 min after completion of colonoscopic examination compared with group II (2.06, SD 1.21 vs 2.14, SD 1.20). However, no significant difference was noted between groups I and II regarding postprocedural pain recorded 1 h after the examination (1.38, SD 0.66 vs 1.37, SD 0.57). Furthermore, we analyzed BMI in relation to loop formation and found that the number of loops was reduced in obese patients (Table 5).

**Table 5:** Comparison of loop formation with BMI in both groups of patients

BMI		Group	Loops [mean]	Loops SD±
<17	Severely underweight	I	0	0
		II	3	0
17–18.49	Underweight	I	2	1.10
		II	3	1.13
18.5–24.99	Normal (healthy weight)	I	1.37	1.41
		II	1.86	0
25–29.99	Overweight	I	1.40	1.05
		II	1.71	1.18
30–34.99	Obese class I	I	1.24	0.97
		II	1.52	0.93
35–39.99	Obese class II	I	1.18	0.87
		II	1.09	0.70
>40	Obese class III	I	0.75	1.50
		II	0.67	0.58

## Discussion

The colonoscopic insertion technique remains one of the most difficult endoscopic procedures to master, and the development of a new colonoscope that is easier to insert is anxiously awaited, especially a colonoscope that can be inserted into the cecum without patient discomfort. Non-sedated colonoscopy may be an uncomfortable or painful examination. It is very important for the colonoscopist to understand the structure of the endoscope during its insertion to successfully accomplish cecal intubation with minimal pain. It has been previously suggested that variable stiffness colonoscopes offer an advantage compared with standard adult colonoscopes given its smaller diameter and increased flexibility [9–11]. Therefore, the purpose of our study was to evaluate whether the RIT colonoscopies could further facilitate the practice of colonoscopic examination performed without analgesia.

We did not find publications evaluating the learning curve to achieve competency at colonoscopy with the use of RIT. Theoretically the learning curve could affect the obtained results; however, the participation of experienced endoscopists with comparable experience and knowledge of the different types of endoscopic instruments eliminates the mistake that could change the results of the study.

In our study, we observed no differences between the two types of colonoscope (RIT vs VSC) regarding cecal intubation rate. This result was expected for the following two reasons. First, the total cecal intubation rate is very high in our endoscopic clinic because most colonoscopies without sedation are performed by experienced endoscopists [12]. Moreover, in the control group, colonoscopies equipped with variable stiffness were used because these endoscopes were previously demonstrated to improve the percentage of cecal intubation [13]. This result is consistent with the previous reports of skilled technical colonoscopists [14–16].

An important finding of this study was that the time needed to reach the cecum was reduced in the RIT endoscope group compared with the VSC group. This finding has also been reported in previously published studies [13, 17]. The time differences obtained in our study were small and therefore of doubtful clinical relevance. Nevertheless, the differences were statistically significant.

**Fig. 2:**
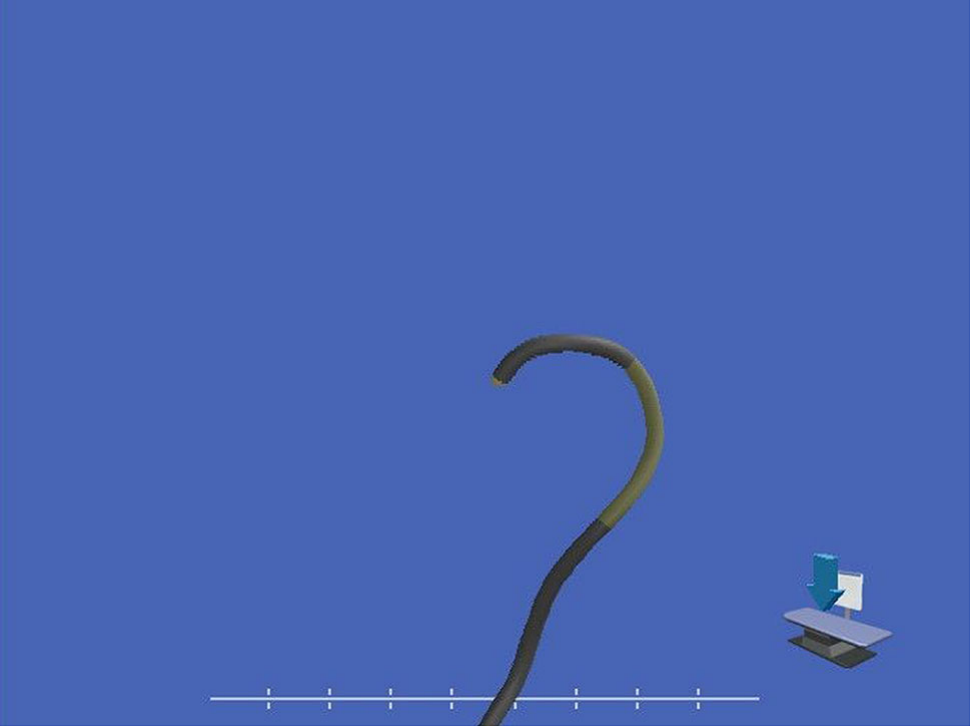
MEI: mild endoscope passage through the splenic flexure with use of RIT

**Fig. 3:**
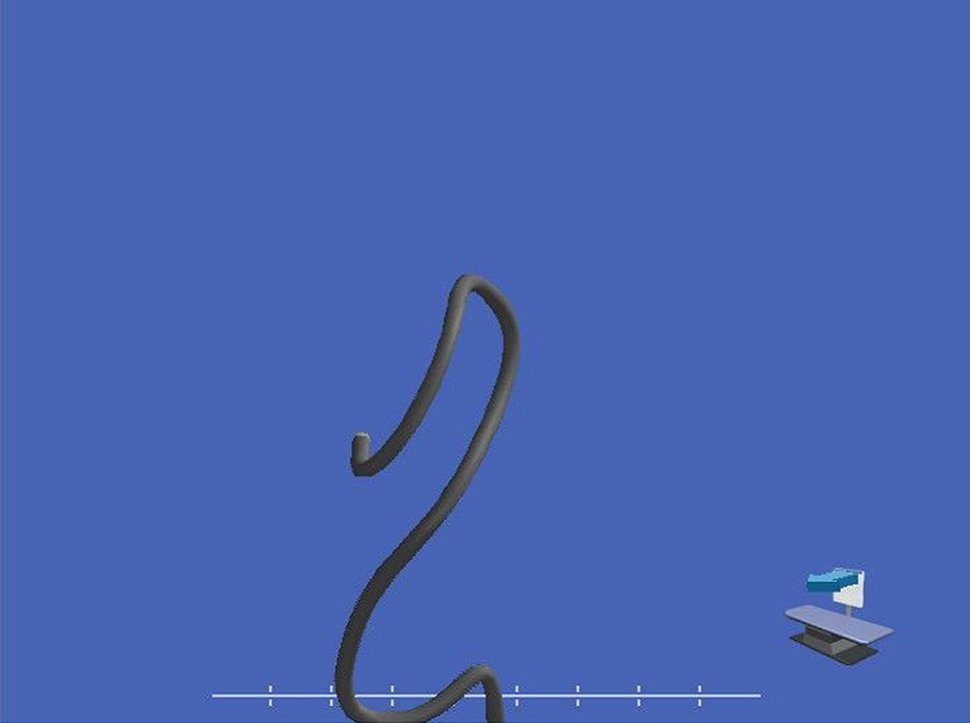
MEI: acute angle of endoscope passage through the splenic flexure using conventional technology (flexure under tension)

One of the major causes of pain during colonoscopy involves the looping of the instrument during insertion through the sigmoid colon, which causes discomfort by stretching the mesentery [18–20]. The number of undesired loops in the shaft of a flexible scope in our study was significantly reduced when the RIT endoscope was used, and less manual pressure to the abdomen was required. This result is likely because the secondary bending section of the endoscope bends only passively and is extremely flexible. This feature is useful in the presence of sharply angulated sigmoid looping. In conventional colonoscopes, when the scope passes through a sharp flexure in the colon, the force applied by the physician when inserting the scope can sometimes directly push up the wall of the colon because the distal end of the scope bends with a small radius—commonly known as the stick phenomenon. The bending function is useful for preventing the stick phenomenon, which causes severe pain for patients during colonoscopic insertion in splenic or hepatic flexures [21]. Reduced loop formation and auxiliary maneuvers when using RIT contribute to a reduction in patient discomfort. We demonstrated that the mean pain score, as rated by the patients, was significantly reduced in patients undergoing unsedated colonoscopy with RIT compared with VSC. This reduction in pain could be attributed to less stretching of the sigmoid colon loops by the flexible intubation tube acquired from the most flexible mode, thereby reducing both the number of auxiliary maneuvers applied (PB and HFT combined) and the recurrent loop formation by the stiffened colonoscope using the stiffest mode (VS) (Figs. 2 and 3).

Abdominal pain during colonoscopy can be affected by multiple factors. Loops caused by the colonoscope may lead to mesenteric stretching that is often associated with discomfort or pain. In addition, endoscope passage through angled colonic flexures, duration of the study, aggressive movements of the endoscope, and gas used for bowel insufflation have also significant impact. Intestinal wall tension is sensible during examination, and for a short time afterward, while the procedure time and gas pressure left in the intestine appear to have a greater effect on the persistence of the postprocedural pain. Thus the application of carbon dioxide insufflation instead of air reduces pain and bloating not only during but also after colonoscopy [22]. This is reflected in our results, where it has been shown that facilitation of the endoscope passage to the cecum due to RIT usage significantly reduces pain during examination and within a short period afterward. The association between body weight and the technical difficulty in achieving CI during colonoscopy has been a topic of debate. Conflicting evidence suggests that both lean and obese subjects present a challenge to the endoscopist during colonoscopy [23–26]. Obesity has been independently associated with poor bowel preparation, which can subsequently lead to a difficult and prolonged colonoscopy. In our study, a lower BMI was an independent factor associated with significant discomfort during colonoscopy. It is possible that the low muscle content of a low-BMI patient may predispose to loop formation and patient intolerance. Our study revealed that the number of loops formed during the insertion of the endoscope was greater in slender low-BMI patients; however, RIT did not alter patient tolerance.

In addition, we must emphasize the safety of the RIT endoscope because no complications associated with its use were noted in the study.

The limitations of this study are the necessity to purchase RIT-equipped endoscopes which are more expensive than the earlier generations. Another limitation is that all endoscopists must be familiar with the skillful use of variable stiffness technology. A criticism of the study is also that, due to the nature of the test, it could not be double-blinded. The endoscopists knew with which colonoscope they were performing the test as it was simply impossible to hide the type of endoscope from them. It should be emphasized that only experienced endoscopists participated in this study and their skillfulness is proved by the efficient cecal intubation time in the control group, which is significantly shorter as compared to the literature [27]. This was certainly influenced by the routine use of magnetic positioning system and the exclusion from the study patients after prior abdominal surgery.

In conclusion, RIT combines three unique technologies: high-force transmission (HFT), passive bending (PB), and variable stiffness. These technologies improve endoscope insertability and ergonomics. Through the use of RIT, the endoscope offers improved operator control when maneuvering and moves more easily through the colon. New RIT instruments allow a favorable colonoscopy with regard to completeness and time required for cecum intubation and significantly reduces discomfort in unsedated patients. These features suggest that RIT is the preferred improvement for unsedated patients undergoing total colonoscopy regardless of the skills of the examiner who can appropriately manipulate this novel device. The use of this technology should also facilitate to conduct colonoscopy under sedation, making it easier to pass the endoscope through the intestine and reduce cecal intubation time.
